# The detection of specific hypermethylated *WIF1* and *NPY* genes in circulating DNA by crystal digital PCR™ is a powerful new tool for colorectal cancer diagnosis and screening

**DOI:** 10.1186/s12885-021-08816-2

**Published:** 2021-10-10

**Authors:** Alexis Overs, Mylène Flammang, Eric Hervouet, Laurent Bermont, Jean-Luc Pretet, Borg Christophe, Zohair Selmani

**Affiliations:** 1 Department of Oncobiology, University Hospital of Besançon, Besançon, France; 2 INSERM, UMR1098, UFC, Besançon, France; 3 EA3181, UBFC, UFC, Besançon, France; 4 Department of Medical Oncology, University Hospital of Besançon, Besançon, France

**Keywords:** Colorectal cancer, Liquid biopsy, Epigenetics, DNA methylation, *NPY*, *WIF1*, Digital PCR

## Abstract

**Background:**

In oncology, liquid biopsy is of major relevance from theranostic point of view. The searching for mutations in circulating tumor DNA (ctDNA) in case of colorectal cancers (CRCs) allows the optimization of patient care. In this context, independent of mutation status biomarkers are required for its detection to confirm the presence of ctDNA in liquid biopsies. Indeed, the hypermethylation of *NPY* and *WIF1* genes appear to be an ideal biomarker for the specific detection of ctDNA in CRCs. The objective of this work is to develop the research of hypermethylation of *NPY* and *WIF1* by Crystal Digital PCR™ for the detection of ctDNA in CRCs.

**Methods:**

Detection of hypermethylated *NPY* and *WIF1* was developed on Cristal digital PCR™. Biological validation was performed from a local cohort of 22 liquid biopsies and 23 tissue samples from patients with CRC. These patients were treated at the University Hospital of Besancon (France).

**Results:**

The local cohort study confirmed that *NPY* and *WIF1* were significantly hypermethylated in tumor tissues compared to adjacent non-tumor tissues (*WIF1 p* < 0.001; *NPY p* < 0.001; non-parametric Wilcoxon paired-series test). Histological characteristics, tumor stages or mutation status were not correlated to the methylation profiles. On the other hand, hypermethylation of *NPY* or *WIF1* in liquid biopsy had a 95.5% [95%CI 77–100%] sensitivity and 100% [95%CI 69–100%] specificity.

**Conclusion:**

Using Crystal digital PCR™, this study shows that hypermethylation of *NPY* and *WIF1* are constant specific biomarkers of CRCs regardless of a potential role in carcinogenesis.

**Supplementary Information:**

The online version contains supplementary material available at 10.1186/s12885-021-08816-2.

## Introduction

Colorectal cancer (CRC) is the third most common cancer worldwide with more than one million new cases diagnosed every year. The development of new chemotherapies, especially cancer personalized therapies, has improved outcomes of patients with CRC. The effectiveness of targeted therapies is based on mutational profiles of RAS/MAPK pathway genes [1]. These mutations are typically sought at the time of diagnosis from a cancer tissue biopsy. However, in cases of a non-feasible biopsy, this search can be performed on circulating tumor DNA (ctDNA). Liquid biopsy is an increasingly common oncology test for the diagnosis of cancer and follow-up of treatments. The search for ctDNA mutations is mainly used in a theranostics approach, particularly in lung and colorectal cancers [2]. The liquid biopsy is a non-invasive approach and can be repeated over time to perform dynamic monitoring of tumors. Unfortunately, this theranostic approach depends on the fluctuant release of tumoral DNA in the vascular compartment [3].

Next Generation Sequencing (NGS) strategies are now commonly used for the detection of mutations on ctDNA [4]. Advanced quantitative technologies, such as the digital PCR (dPCR), have been developed to increase sensitivity of detection [5]. The dPCR amplifies millions of individual DNA fragments using thousands of water-in-oil droplets. This compartmentalization increases the detection sensitivity, and is especially adapted for mutations present in low concentration. If no mutations are detected in liquid biopsy, discrimination between an unmutated profile and an absence of ctDNA in the plasma sample is not possible. To address this issue, high sensitivity tumor-specific epigenetic biomarkers have been identified to assert presence of ctDNA [6, 7]. DNA methylation is the most studied epigenetic mechanism in this respect [8].

Indeed, the tumor cell epigenome associates global hypomethylation [9, 10] interspersed with hypermethylated specific regions such as promoters of tumor suppressor genes, and correlated with a decreased expression [11]. These modifications happen in tumorigenesis and many studies are looking at these new epigenetic markers [11, 12].

Based on Roperch and *al* [13]. and Garrigou and *al* [14]. studies, DNA hypermethylation of *NPY* (Neuropeptide Y) and *WIF1* (Wnt inhibitory factor 1) appears as a specific marker of ctDNA in CRCs. Hypermethylation of *NPY* and *WIF1* is found in 100% of CRCs [13, 14], while the presence of a defined mutation is inconstant in CRCs. For instance, a mutation of *KRAS* is only found in 40% of CRCs and therefore cannot be used as a biomarker for the presence of CRCs ctDNA. Thus, hypermethylation of *NPY* and *WIF1* appears to be a better biomarker [6].

In the present study, we adapted a droplet-based dPCR protocol previously described [13, 14] in the Naica Crystal Digital PCR system™ (Stilla Technonologies, Villejuif, France) in order to investigate the hypermethylation of the *NPY* and *WIF1* in CRC tissues or ctDNA. The objective is to determine whether the hypermethylation of *NPY* and *WIF1* is a specific biomarker of CRC in liquid biopsy by Crystal Digital PCR™ and could be used for routine diagnosis, recurrence and treatment follow-up.

## Materials and methods

### Patients

Digital PCR analyses were conducted on 22 blood and 23 tissue samples from patients with CRC treated at the University Hospital of Besancon (France) (Table 1). Before inclusion all patients provided written informed consenting to the use of their clinical, biological and demographic data for research purposes. Samples were preserved in the framework of the “Tumorothèque Régionale de Franche-Comté”. This scientific board has an authority to approve human studies. And blood samples from patients without any oncologic background (considered as control group) were collected at the “Etablissement Français du sang”. These samples were blood donations.

**Table 1:** Demographic, anatomopathological and biological data of the cohort. Twenty-three colorectal cancer tumor tissues were included and 22 circulating tumor DNA. For 11 of the tumor tissues, non-tumor tissues close to the tumor were analyzed. The anatomopathological and biological results come from analyses carried out as part of patient management

	Tumor/non-tumor tissues pairs (***n*** = 11)	Tumor tissues (***n*** = 12)	ctDNA (***n =*** 22)
**Sex, n (%)**			
Men	5 (45%)	6 (50%)	16 (73%)
Women	6 (55%)	6 (50%)	6 (27%)
**Age in years, mean (min-max)**			
	60 (44–79)	75 (55–84)	63 (36–82)
**Location of the tumor, n (%)**			
Duodenum	/	1 (8%)	/
Cecum	2 (18%)	2 (17%)	2 (9%)
Right colon	2 (18%)	6 (50%)	3 (13%)
Transverse colon	/	2 (17%)	1 (5%)
Left colon	5 (46%)	/	11 (50%)
Rectosigmoid junction	1 (9%)	1 (8%)	1 (5%)
Rectum	1 (9%)	/	4 (18%)
**Conservation, n (%)**			
FFPE	8 (72%)	12 (100%)	/
Freezing	3 (28%)	/	/
**Histology, n (%)**			
ADC	11 (100%)	12 (100%)	19 (86%)
Tubular adenoma	/	/	1 (5%)
NA	/	/	2 (9%)
**Stage, n (%)**			
I	1 (9%)	2 (17%)	2 (9%)
II	4 (37%)	5 (42%)	/
III	3 (27%)	2 (17%)	/
IV	3 (27%)	2 (17%)	20 (91%)
ND	/	1 (8%)	/
**Microsatellite stability, n (%)**			
MSS	7 (64%)	2 (17%)	16 (72%)
MSI	3 (27%)	10 (83%)	1 (5%)
NA	1 (9%)	/	5 (23%)
**Mutational status, n (%)**			
*KRAS* mutation	3 (27%)	3 (25%)	7 (32%)
*NRAS* mutation	/	/	2 (9%)
*BRAF* mutation	3 (27%)	4 (33%)	1 (5%)
Other mutations	/	/	1 (5%)
No mutation	1 (9%)	5 (42%)	11 (50%)
NA	4 (36%)	/	/
***MLH1*** **methylation, n (%)**			
Presence (≥ 5%)	3 (27%)	9 (75%)	/
Absence (< 5%)	/	3 (25%)	/
NA	8 (73%)	/	22 (100%)

*ADC* adenocarcinoma, *ctDNA* circulating tumor DNA, *FFPE* formalin-fixed paraffin-embedded, *MSS* microsatellite stable, *MSI* microsatellite instability, *NA* not available

### DNA isolation and bisulfite modification

Tumors DNA was extracted from frozen biopsies and FFPE samples. In EDTA collected blood samples were pre-treated to obtain supernatants which were stored at − 80 °C. Circulating cell-free DNA (cfDNA) was extracted from 4 mL to 6 mL of plasma using the QIAamp® Circulating Nucleic Acid kit (Qiagen®, Hilden, Germany) according to the manufacturer’s protocol and resuspended in 50 μL of buffer. The quantity of DNA was mesured by Qubit 2.0 fluorometer (Invitrogen®, Life Technologies) and were obtain between 1 to 240 ng/μL (mea*n* = 22.6 ng/μL).

For all samples, bisulfite treatment was performed to transform unmethylated cytosine into thymidine without changing methylated cytosine, by EZ DNA Methylation kit® (Zymo Research) for DNA concentration of 1 ng/μL.

Mutation status Analysis by NGS, microsatellite phenotype and *MLH1* promoter methylation by pyrosequencing were performed as part of patient management. After bisulfite treatment of tumor DNA, 5 μL of the bisulfite-treated DNA solution was analyzed by a pyrosequencing technique according to the PyroMark™ Q24 CpG MLH1 procedure (Qiagen®, Hilden, Germany). The analyzed promoter sequences correspond to the proximal region, − 209 to − 181, relative to the transcription start site of *hMLH1* gene.

### Development of the digital PCR analysis

**Fig. 1:**
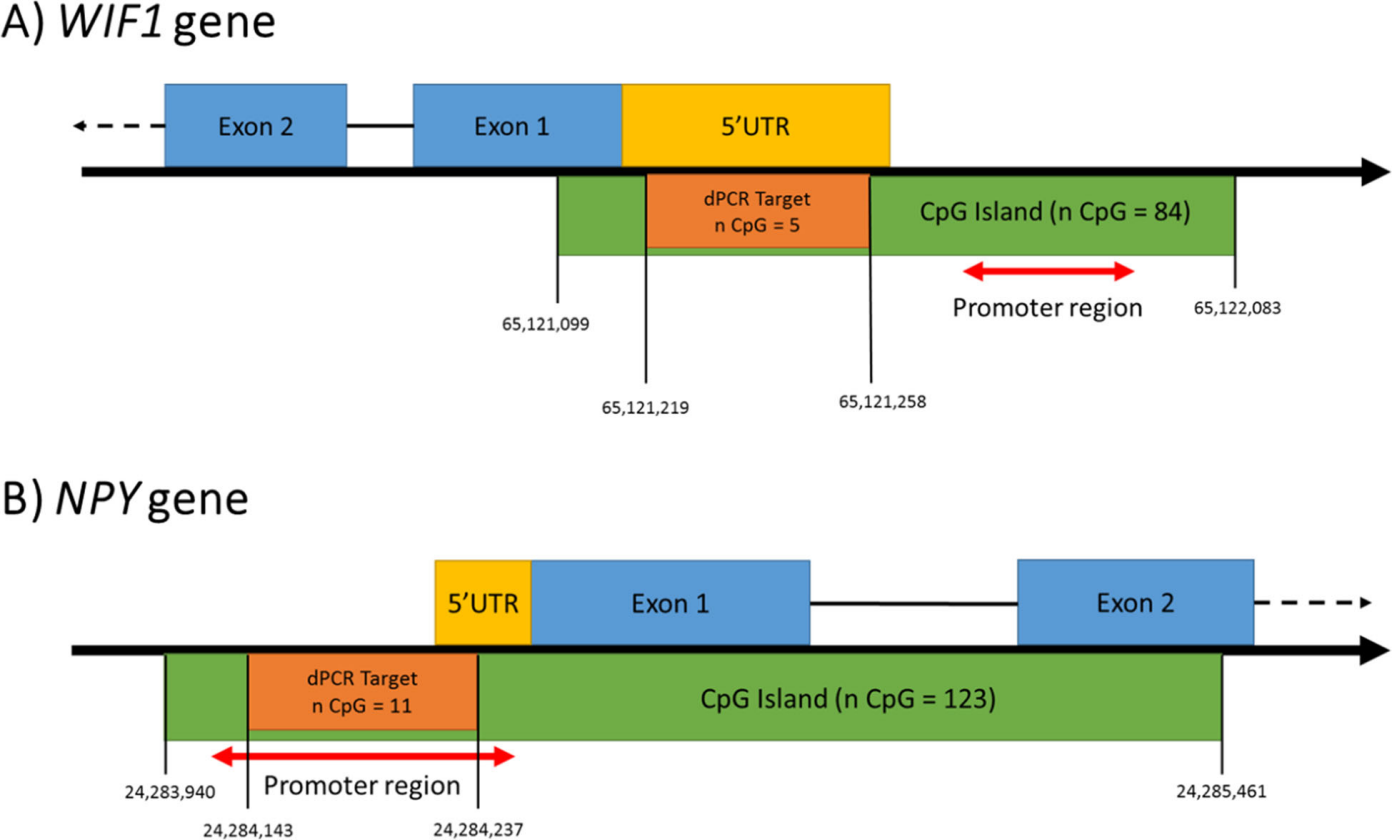
Genomic environment of the dPCR target associated with *WIF1* and *NPY.* A) *WIF1* is located on the reverse strand of the 12th chromosome’s long arm (12q14.3). The 5′ region of *WIF1* is covered by a 1 kb wide CpG island that contains 84 CpG. Within this CpG island is the 67 bases wide dPCR target that contains 5 CpG. The target is entirely contained within the 5’UTR of *WIF1* and does not overlap the promoter of *WIF1* (chr12: 65,121,532-65,121,731) (Encode: EH38E16200601). B) *NPY* is located on the short arm of the 7th chromosome (7p15.3). The 5′ region of NPY is covered by a 1,6 kb wide CpG island that contains 123 CpG. Within this CpG island is the 96 bases wide dPCR target that contains 11 CpG. It partially overlaps with the 5’UTR of *NPY* and is entirely contained within the promoter of *NPY* (chr7: 24,283,952-24,284,290) (Encode: EH2540595). Illustration not to scale for clarity.

An aberrant hypermethylation of *NPY* and *WIF1* genes has been described in CRCs. We developed a (previously described) 2-panel assay targeting these biomarkers previously described on a Naica Crystal Digital PCR system™ (Stilla Technonologies, Villejuif, France). After bisulfite conversion, a volume of 5 μL of DNA extract was assembled in 20 μL PCR mixtures using 1 X PerfeCTa Multiplex qPCR ToughMix (Quanta Biosciences, Gaithersburg, MD, USA), 100 nM Fluorescein, 1 μM each primer, 250 nM each hydrolysis probe. In Table 2, probe and primer sequences were previously designed by Garrigou et al. [14] (Fig. 1). But we developed Crystal digital PCR™ specific conditions by 95 °C for 5 min, followed by 50 cycles of 95 °C for 15 s and 57 °C for 10 s.

**Table 2:** Probe and primer sequences for *NPY* and *WIF1* genes

Gene	Oligo type	Sequence	Fluorophore	Tm (°C)
***NPY***	Forward primer	5′ CGCGGCGAGGAAGTTTTATA 3′	/	58
	Reverse primer	5′ ATACTATCGAACGAACGTCTCCG 3′	/	64
	Probe	5′ CGCGATTCGTTTTTTGTA 3’	Cyanine 5	47.8
***WIF1***	Forward primer	5′ GAGGGAGTTGTAGCGTAGTAGAGTATTTG 3’	/	58
	Reverse primer	5′ AAAACTCCTCGTACCGCACCTA 3’	/	54
	Probe	5′ CGGCGTTAGGTTGC 3’	Cyanine 3	56

### Data analysis

The droplet identification and fluorescence measurements in each detection channel were performed using Stilla’s Crystal Miner® software. Spill-over compensation was defined and applied. Gating of positive and negative droplet clusters was performed.

Transcriptional impact of hypermethylation of *NPY* and *WIF1* was evaluated on the TCGA-COAD data. Transcription data were normalized with Deseq2 [15] and gene expression was compared between non-tumor (*n* = 41) and tumor samples (*n* = 480) using bilateral Student test.

### Statistical analysis

An analysis of the difference in methylation between tumor and healthy tissue was conducted using the non-parametric Wilcoxon test. The difference in methylation between the 2 groups was determined using a non-parametric Mann-Whitney test; the analysis of more than 2 groups was performed using the non-parametric Kruskal-Wallis test. Spearman’s nonparametric test was used for correlation research. Statistical analyses were performed using GraphPad (GraphPad Software Inc., San Diego, CA). An uncertainty of 5% was defined for each of the tests and a *p*-value < 0.05 was considered statistically significant.

The required numbers of subjects (RNS) per groups were computed with the software R v4.0.2 using the observed means and standard deviations in our cohort and the usual statistical parameters (a significance level of 0.05 and a power of 0.90). Grouping by tumor/non-tumor, tstandard deviations were significantly different, therefore the ANOVA test was used. This estimation shows that an important difference of the positive droplets number between tumor and non-tumor. For the tumoral status (tumor vs non-tumor), the estimated RNS was 10 samples for *WIF1* and 16 samples for *NPY*. Using a Student test for the liquid biopsies analyzes, the RNS were 10 samples for *NPY* and 9 samples for *WIF1*.

In our study, 23 tumor tissues and 22 bloods samples were analysed with powerful significativity (*p* < 0.001).

## Results

### Detection of *NPY* and *WIF1* methylation by crystal digital PCR™

A digital PCR technique has been developed on the Naica Crystal Digital PCR system™ (Stilla Technonologies, Villejuif, France) for the detection of *NPY* and *WIF1* genes’ methylation.

A limit of blank (LOB) was calculated for the two detection channels allocated to Cyanine 5 and Cyanine 3 for *NPY* and *WIF1* respectively. A total of 12 experiments were performed with unmethylated DNA quantified at 0.2 ng/μL. Garrigou et al. [14] showed that the rate of false positive droplets is independent of the total amount of DNA. The LOB with the confidence level (1-α) was defined as the maximum number of false positive events that are plausible with a 1-α level probability (95% for risk α = 5%). The number of false positive droplets was recorded to targeted channel detection. The LOB was set as one false positive droplet for *NPY* and five for *WIF1* promoters’ methylation.

**Fig. 2:**
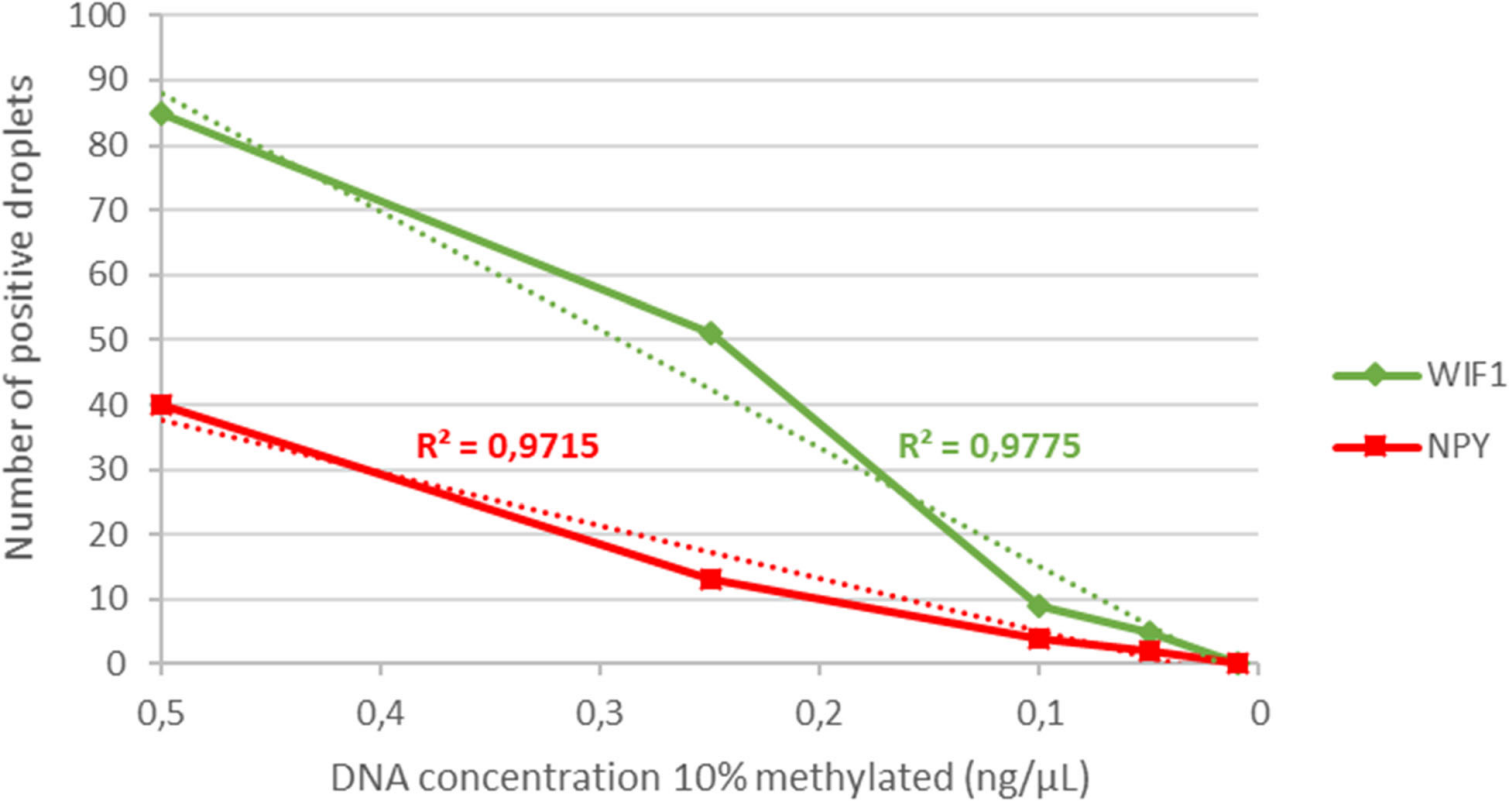
Detection of the methylation status of *NPY* and *WIF1* according to the concentration of DNA methylated at 10%. The number of positive droplets is expressed according to the five DNA concentrations: 0.5, 0.25, 0.1, 0.05 and 0.01 ng/μL. The correlation coefficients show the high sensitivity of the technique for low concentrations

A dilution test was performed in order to assess the detection sensibility of the technique. Five concentrations of a fully methylated control DNA (EpiTect Qiagen®, Hilden, Germany) at 10% have been tested: 0.5 ng/μL, 0.25 ng/μL, 0.1 ng/μL, 0.05 ng/μL, and 0.01 ng/μL (Fig. 2). For a concentration of 0.05 ng/μL with a percentage of DNA methylated at 10%, the developed technique was able to detect the methylation of the *NPY* genes (*R*^2^ = 0.9715) and *WIF1* (*R*^2^ = 0.9775). These results show the high sensibility of this method. The difference in the level of detection between the two targets can be explained by the difference in the numbers of CpG analyzed by our technique (Fig. 1). Indeed, the methylation profile of *NPY* is more restrictive because 11 CpG must be methylated for their detection. The detection of *WIF1* applies to a region of five CpG.

### Validation on local cohort

#### Characteristics of the patient population

A cohort of 45 patients (22 blood and 23 tissue samples) with CRC was included in the study (Table 1). The average age of patients was 62 years which is similar to the average age of CRC diagnosis [16]. In the first instance, DNA extractions from 11 tumors/non-tumor tissues pairs were included. Three of those pairs derived from frozen tissue samples and 8 from formalin-fixed paraffin-embedded (FFPE) tissue samples. Tumor tissues corresponded to primary tumors biopsies. Anatomopathological data show that all tumors are adenocarcinomas and that almost half of them are located in the left colon (46%). On the 23 CRCs DNA analyzed, 59% show microsatellite instability (MSI), of which 52% present a MLH1 promoter methylation, and almost half (56%) arbor a *KRAS* (26%) or *BRAF* (30%) mutation. In parallel, 22 plasma DNA extracts from CRC patients, mostly stage IV (91%), were included and somatic mutation initially detected in tumor DNA was found for half of the ctDNA samples (*n* = 11, 50%). The plasma of 10 patients was also analyzed as a control group (Mean age: 49 years (min-max: 21-65y), 50% are female) (Supplementary data).

#### CRC patient tissues detection of *NPY* and *WIF1* DNA methylation

**Fig. 3:**
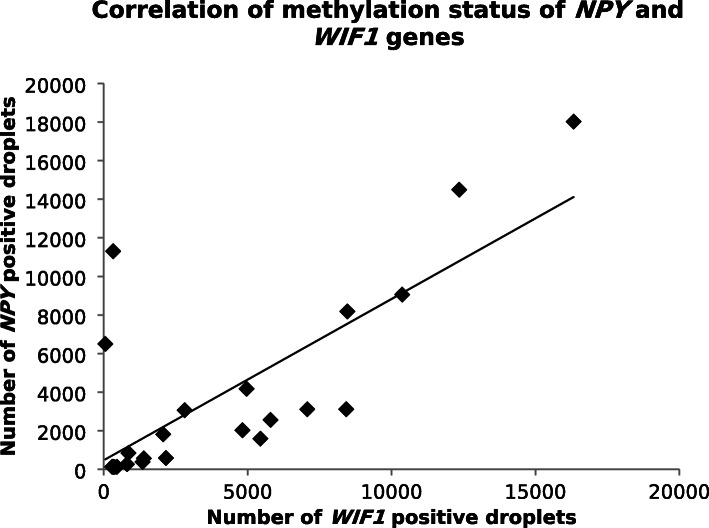
Correlation of *NPY* and *WIF1* gene methylation status in colorectal tumor tissues. (*n* = 23, *R*^2^ = 0.56, *p* = 0.0016, non-parametric Spearman’s test)

Using Crystal Digital PCR™, the *NPY* and *WIF1* hypermethylation testing was performed on the 23 tumor tissues from CRC patients. Specific hypermethylation of *NPY* and *WIF1* was observed for all samples (100%). A significant correlation was showed between the number of positive droplets for both genes (*R*^2^ = 0.56, *p* = 0.0016, non-parametric Spearman’s test) (Fig. 3). With non-parametric Spearman’s test, the number of positive droplets was also correlated with the concentration of DNA extracted for *WIF1* (*R*^2^ = 0.436, *p* = 0.0377) and for *NPY* (*R*^2^ = 0.809, *p* < 0.0001). For control group, 11 CRC adjacent non-tumor tissues were used.

**Fig. 4:**
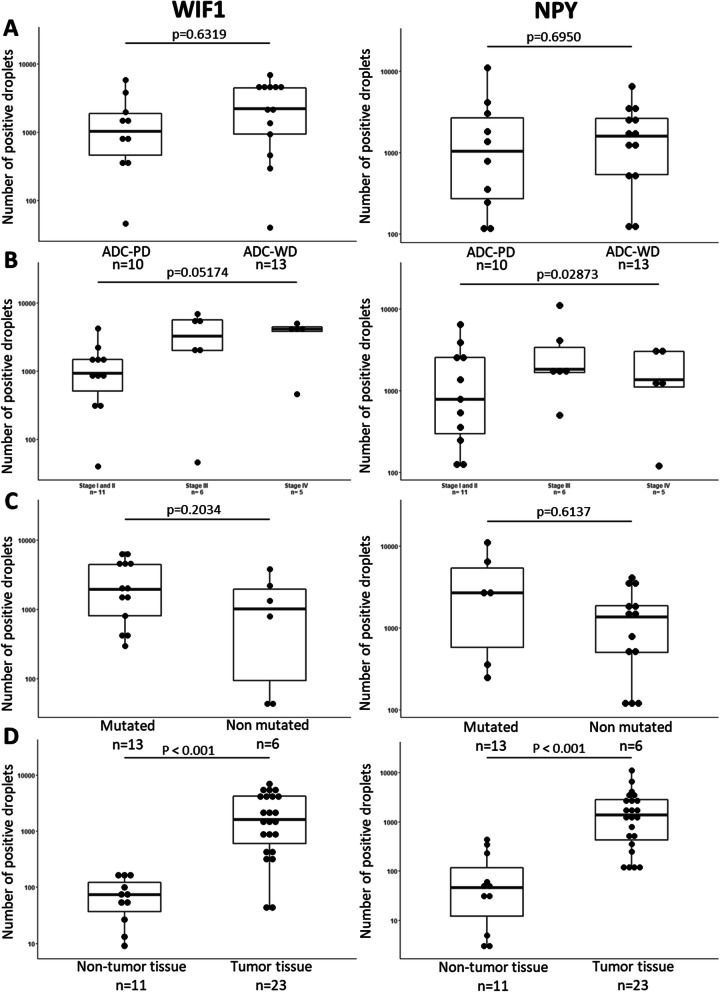
Comparison of methylation according to histology, tumor stages and mutation status. Comparison of the methylation of *WIF1* and *NPY* genes according to A) the histology of the tumor (ADC-WD or ADC-PD). Non-parametric Mann-Withney test. B) Tumor stage (stage I and II, III or IV). Non-parametric Kruskal-Wallis test. C) The mutated or non-mutated status of the tumor. Non-parametric Mann-Whitney test. D) Tumor tissues compared to the adjacent healthy tissues. Non-parametric Mann-Withney test in paired series. *ADC-WD: well-differentiated adenocarcinoma, ADC-PD: partially differentiated adenocarcinoma*

A comparison of the number of positive droplets was performed according to histology (partially or well differentiated adenocarcinoma) and tumor stage (I and II, III or IV). No significant difference was found between the methylation profile and tumor histology (*p* = 0.6950; for *NPY p* = 0.6319 for *WIF1*; non-parametric Kruskal-Wallis test, *n* = 21) (Fig. 4A). The same absence is observed with the tumor stage (*p* = 0.2873 for *NPY*; *p* = 0.0517 for *WIF1*; non-parametric Kruskal-Wallis test, *n* = 22) (Fig. 4B).

The mutation tumor status as well as its microsatellite status (MSS or MSI) has also been compared with methylation profiles. No significant difference was found for mutation status (*p* = 0.6137 for *NPY*; *p* = 0.2034 for *WIF1*; non-parametric Mann-Whitney test, *n* = 18) (Fig. 4C) nor for microsatellite status (*p* = 0.1439 for *NPY*; *p* = 0.8860 for *WIF1*; non-parametric Mann-Whitney test, *n* = 22). The percentage of *MLH1* promoters’ methylation was not correlated with *WIF1* and *NPY* promoters’ methylation (*NPY*: *R*^2^ = 0.07; *WIF1*: *R*^2^ = 0.45, *p* = 0.0972; *p* = 0.8124; non-parametric Spaerman’s test, *n* = 15).

Importantly, significant hypermethylation of both genes was demonstrated in tumor tissues compared to adjacent non-tumor tissues (*NPY p* = 0.001; *WIF1 p* = 0.002; non-parametric Wilcoxon paired-series test) (Fig. 4D). The *NPY* and *WIF1* methylation testing was performed on the 11 pairs of tumor/non-tumor tissue adjacent to the tumor. The *WIF1* gene mean positive droplet number for tumor and non-tumor tissue were respectively 6776 and 81. The Area Under Curve (AUC) of the Receiver Operating Characteristic (ROC) curve was 0.937 [0.845–1.000] (Supplementary data 2A). For *NPY* gene, the mean droplet number for tumor and non-tumor tissue were respectively 6021 and 115. The AUC of the ROC curve was 0.979 [0.933–1.000] (Supplementary data 2B). Using the two biomarkers, we obtained an AUC = 1 [1.000–1.000] on our local cohort (Supplementary data 2C).

**Fig. 5:**
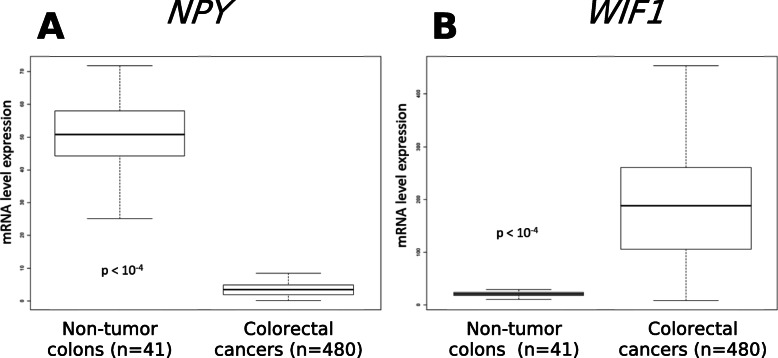
Expression of the *NPY* and *WIF1* mRNA in CRCs tissues and non-tumor tissues. The transcriptomic study of the Cancer Genome Atlas (TCGA) allows the analysis of the levels of mRNA encoded by the genes. **A)** Thus, it is shown that the level of mRNA encoded by the *NPY* gene is significantly lower in colorectal cancers than in healthy tissues (*p* < 10^− 4^). **B)** On the other hand, the level of mRNA encoded by the *WIF1* gene is higher in colorectal cancers than in healthy tissues (*p <* 10^− 4^)

As shown by multivariate Anova analyzes, the *NPY* and *WIF1* methylation are powerful biomarkers of all types of CRCs independently of mutations, MSI and *MLH1* methylation status. Comparing tumor samples and non-tumor colonic tissues, TCGA data were analyzed for *WIF1* and *NPY* transcripts (Fig. 5). The *NPY* transcripts in CRCs are lower than in non-tumor tissues (Fig. 5A). However, transcriptomic analysis shows overexpression of *WIF1* in CRCs (Fig. 5B).

#### CRC patient liquid biopsies detection of *WIF1* and *NPY* DNA methylation

**Fig. 6:**
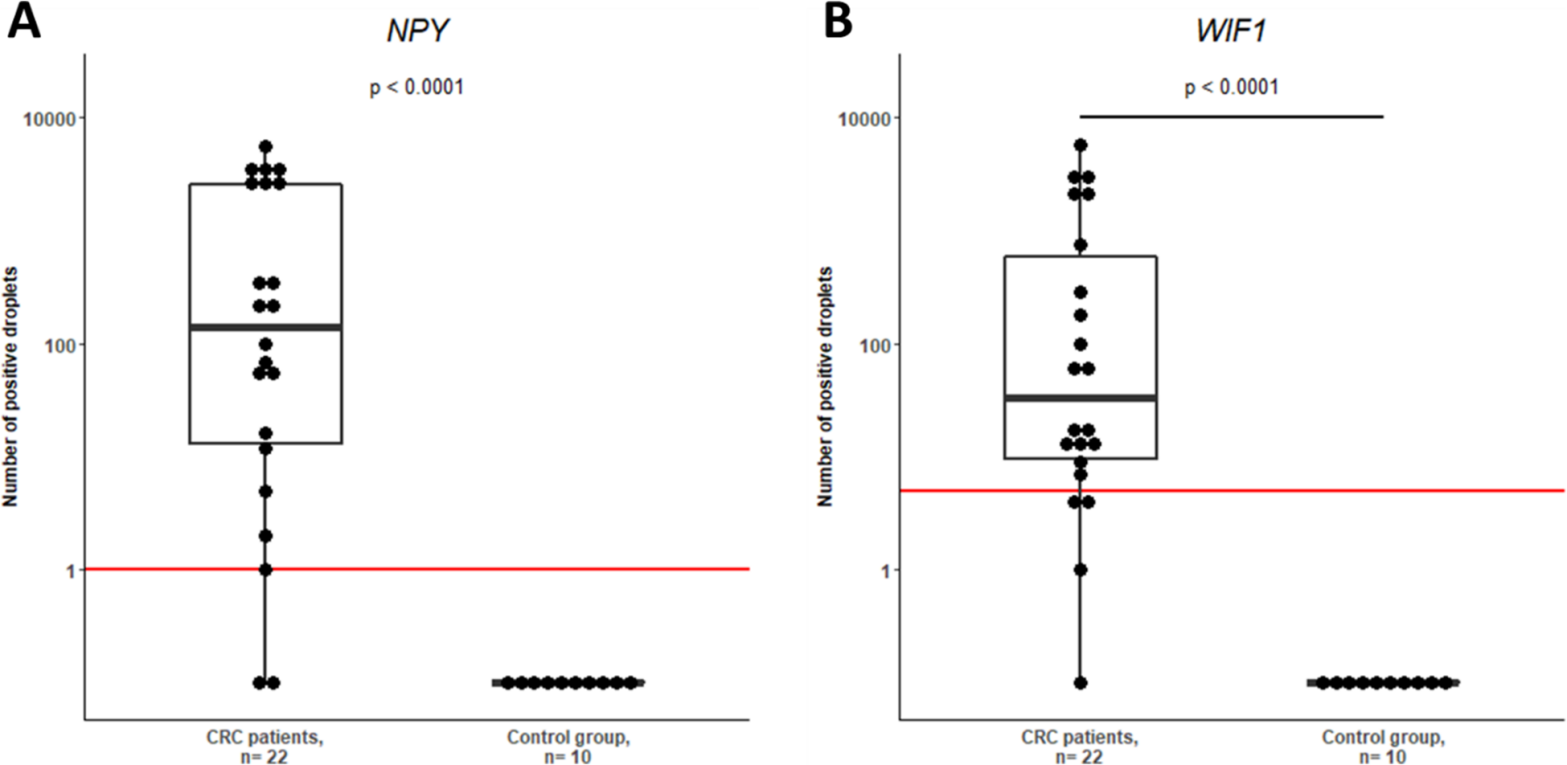
CRCs ctDNA compared to control group in liquid biopsies. Comparison of *NPY* **(A)** and *WIF1* **(B)** hypermethylation in liquid biopsies of patients with a CRC compared to control group by Mann Whitney test. The red line represents the specific threshold of positivity: ≥1 positive droplet for *NPY* and ≥ 5 positive droplets for *WIF1*

In the cohort of 22 total circulating DNA samples, hypermethylation of *NPY* or *WIF1* in liquid biopsy had 95.5% of sensitivity [95% CI, 77 to 100%] and 100% of specificity [95% CI, 69 to 100%]. And hypermethylation of *NPY* or *WIF1* was observed for 95.5% of the extracts, of which 77.3% with methylation for both genes. All patients with stage IV disease were detected (Fig. 6). For one stage I CRC patient, the extract showed no methylation for both the genes. This extract was characterized by a low concentration of total circulating DNA (1.1 ng/μL) and no mutation was detected by NGS either on the plasma extract or on the tissue biopsy, suggesting a disease in the early stages of carcinogenesis. The ten ctDNA in the control group were analyzed and the methylation of the *NPY* and *WIF1* genes was negative (Supplementary data).

A significant correlation was observed between the concentration of total circulating DNA in the plasma extracts and the number of positive droplets (*p* < 0.0001 for *NPY* and *WIF1*; non-parametric Spearman’s test). As expected, the correlation between the number of positive droplets and the percentage of mutation found in NGS was not observed (*p* = 0.0703 for *NPY*; *p* = 0.0787 for *WIF1*; non-parametric Spearman’s test).

## Discussion

### Methylation of *WIF1* and *NPY* in colorectal tissues

In the cohort, 23 primary colorectal tumor tissues were analyzed associated with 11 adjacent non-tumor tissues to measure DNA hypermethylation of *NPY* and *WIF1*, as potential new biomarkers of CRC in liquid biopsies. A significant hypermethylation of *NPY* and *WIF1* in the tumor tissues was demonstrated (*p* < 0.001 for *NPY; p* < 0.001 for *WIF1*) (Fig. 4D). However, a higher than LOB positive droplet counts was found in healthy tissues. This result could be explained by the very high sensitivity of dPCR. Indeed, tissues are considered healthy by microscopic analyzes but some tumor cells, or pre-tumor cells, would be present without any microscopical characteristic. In addition, biopsies of adjacent non-tumor tissues may contain tumor cells and/or ctDNA within their vascularization. This tumor “contamination” of healthy tissue was already found with low positivity [13, 14]. These results are consistent with those of Roperch et al. who set a methylation threshold above this, and considered the threshold of positive result, 25% for *NPY* and 7% for *WIF1*, because non-tumor adjacent tissues also had a low percentage of methylation level [13].

Furthermore, a significant correlation between the number of positive droplets for *NPY* and *WIF1* was found (*R*^2^ = 0.62, *p* = 0.0016), suggesting that methylation of both genes is early and concomitant in carcinogenesis. On the other hand, no significant correlation was observed with the *MLH1* promoter methylation used as a predictive factor of sporadic forms of CRC, but on the other hand found in MSI CRCs. Thus, methylation of the *NPY* and *WIF1* genes appears to be a process independent of mismatch repair genes methylation and suggests mechanisms of systematic methylation, concerning low significance genes for carcinogenesis (as *NPY* or *WIF1*). And a conditioned methylation of some tumor suppressor genes whose repression is necessary for carcinogenesis process. This is confirmed in tumor DNA extracted from a biopsy for which no methylation of the *MLH1* gene promoter was found, whereas methylation of the *NPY* and *WIF1* genes was observed.

Significant difference in the level of methylation was not found according to the stage of the tumor. These results are consistent with constant methylation of both genes in tumor DNA. *NPY* and *WIF1* DNA methylation are powerful specific biomarkers of all types of CRCs.

### Methylation of *NPY* and *WIF1* on circulating tumor DNA

The method has been validated on tissues and then on ctDNA from CRC patients. All analyzed plasma samples from CRC patients were positive for *WIF1* and/or *NPY* methylation except one stage I patient sample (Fig. 6). This negative DNA extract was very low in total DNA (1.1 ng/μL). The absence of ctDNA in this sample is probably the cause of the negative result [6]. These results were also confirmed in preliminary data on Stilla Application Note [17]. Nevertheless, the presence of methylation of *NPY* and *WIF1* genes in all other samples suggests that methylation process occurs is constant in carcinogenesis. Therefore, the detection of this epigenetic process could be a relevant marker for CRCs screening. Thus, this sensitive and non-invasive technique can be an interesting screening tool for CRCs exploration, and especially in advanced stages that require rapid treatment.

### *NPY* and *WIF1*’s role in colorectal carcinogenesis

The hypermethylation of *NPY* promoter in CRCs leads to a strong repression of its transcription (Fig. 1A). The region targeted by our dPCR protocol partially overlaps with the 5’UTR of NPY and is entirely contained within the promoter of *NPY* (Fig. 1B). The genomic colocation with the promoter could explain the negative correlation between the methylation of the dPCR target and the expression of NPY. Nevertheless, the role of *NPY* in the tumorigenesis process is not fully elucidated. In vitro, *NPY* appears to promote tumorigenesis, probably in a neoneurogenesis context in which tumor cells exploit neurotransmitters to generate a pro-tumor environment [18]. *NPY* repression should thus inhibit tumor proliferation. Paradoxically, NPY appears to reduce the invasive potential of tumor cells in vitro [19]. In the CISTROME database with experimental data, we observed that EP300, EZH2, JARID2, RYBP, PAX5, and SUZ12, might bind the *NPY* targeted region [20]. Also, in silico analysis shows that this CpG island could interact with several transcription factors (TF) such as CTCF, EZH2, GLIS2, RAD21, ZFP37, ZBT family (ZBTB20, ZBTB26, ZBTB17, ZBTB11) and ZNF family (ZNF777, ZNF335) [20]. The specific methylation of the dPCR targeted region could inhibit the transcription of *NPY* enhanced by those TF. Currently, only in vitro data are available and the role of *NPY* in CRCs is still to be defined. By the way, Alshalalfa et al have shown, that in the case of prostate cancer, the decrease of NPY appears to be associated with aggressive phenotype and with a high risk of developing metastasis [21].

Amlal et al. showed that estrogen up-regulates NPY receptor (Y1R) expression through estrogen receptor alpha [22] in breast cancer cell lines. Estrogen plays an important role in the up-regulation of Y1R, which in turn regulates estrogen-induced cell proliferation in breast cancer cells. In another model, estrogen significantly decreased NPY secretion in both the mHypoE-42 and mHypoA-2/12 neurons [23]. These findings indicate that the central anorexigenic action of estrogen occurs at least partially through hypothalamic NPY-synthesizing neurons. Estrogen actions on NPY receptor might affect NPY signaling according to genders. In our study, no signicant differences were observed between female and male patients concerning methylation of *NPY* (*p* = 0.055 for non-tumor tissues and *p* = 0.13 for tumor tissues) (Supplementary data 3A). These observations were confirmed by TCGA-databases analyzes (*p* = 0.89 for non-tumor tissues and *p* = 0.69 for tumor tissues) (Supplementary data 3B). We can suppose that gender does not affect the methylation of *NPY* in CRC carcinogenesis.

Concerning the *WIF1*, its repression leads to an overexpression of the Wnt signaling pathway thus promoting cell transformation [24]. However, transcriptomic analysis from the Cancer Genome Atlas (TCGA) shows overexpression of *WIF1* in CRCs (Fig. 5B). The dPCR target is entirely contained within the 5’UTR of *WIF1* but does not overlap the promoter of *WIF1* (Fig. 1A). Thus, *WIF1* is a tumor suppressor gene whose expression should be rather inhibited in tumor tissues. Therefore, regulatory sequences have to be hypermethylated. We could suggest that sequences analysed by dPCR are not implicated in the regulation of WIF1 expression and could result from systematic methylation. Hypermethylation of dPCR targeted *WIF1* region would therefore not lead to repression of the gene. In CISTROME database, *WIF1* hypermethylated region is experimentally associated with some proteins such as EP300, EZH2, HDAC2, HIC1, JARID2, KDM2B, MBD2, PRDM11, RYBP, SMAD2, SPDEF, SRF, SUZ12, ZMYND8, ZNF180, ZNF189 and ZNF483 [20]. In silico analysis shows that CpG region targeted by dPCR could interact with several TF such as CREB1, CTCF, EGR2, EZH2, GLIS2, HIC1, KDM1A, KLF9, KLF16, PATZ1, POLR2A, TBP, ZBTB family (ZBTB8A, ZBTB17, ZBTB26, ZBTB33, ZBTB48), ZEB2, ZFHX2, ZFP69B, ZFP37ZSCAN21 and ZNF family (ZNF398, ZNF335, ZNF341, ZNF501, ZNF513, ZNF600, ZNF692, ZNF777, ZNF792) [20]. Feng et al demonstrates that miR-590-3p regulates colon cancer progression via *WIF1* which suggests that miR-590-3p may be a promising candidate for therapeutic applications in colon cancer treatment [25]. In nasopharyngeal cancers and gastric carcinoma cell lines the promoters of *WIF1* is hypermethylated, and his expression is regulated by miR-BART19-3p [26]. Also the miR-552-5p promoted osteosarcoma development and progression by inhibiting *WIF1* [27]. These studies show that *WIF1* could be highly regulated by post-transcriptional factors. These data only provide information on the level of mRNA expression but not the protein functionality.

Thus, the hypermethylated promoters of *NPY* and *WIF1* are specific early markers of colorectal cancers but their roles in CRCS carcinogenesis are not clearly established.

### Limitations of the study

The size of our cohort is sufficient to demonstrate the efficiency of our technique for the detection of *NPY* and *WIF1* methylation status. However, it is difficult to make subgroup comparisons. Nevertheless, our study confirms previous studies results suggesting that methylation of one or both genes seems to be a relevant biomarker to detect the presence of ctDNA in plasma liquid biopsies. Our study is robust and highlights an original and powerful technique in the detection of specific methylation profile of CRC. Roperch and *al* [13]. tested 161 sera from patients with normal colonoscopy using Methylation Specific PCR. They showed a specificity of 80 and 95% for *NPY* and *WIF1* respectively. Garrigou et al. analyzed 46 plasmas from non-cancer patients with their dPCR technique. Only 3 patients had a higher than the LOB droplet count for the *NPY* gene, i.e. a specificity of 93%. The specificity of the *WIF1* gene was 100% [14].

Many exogenous factors known to modulate DNA methylation were not included in our study. Indeed, many links between lifestyle and epigenetic modifications have been shown [28]. For example, tobacco [28] or alcohol [29] consumption have been shown to modulate DNA methylation. The comparison between consumers and non-consumers could help to understand if by modifying the methylation profiles, the consumption of tobacco or alcohol, could generate false positive results in our technical approach.

## Supplementary Information

**Additional file 1.**

## Data Availability

The datasets generated and analyzed during this study (birthdate, admission date, discharge date, date of death …), are available from the corresponding author on reasonable request.

## Abbreviations

ADC: Adenocarcinoma
ADC-PD: Partially differentiated adenocarcinoma
ADC-WD: Well-differentiated adenocarcinoma
AUC: Area under the curve
CRC: Colorectal cancer
ctDNA: Circulating tumor DNA
dPCR: Digital Polymerase chain reaction
FFPE: Formalin-fixed paraffin-embedded
LOB: Limit of blank
MSS: Microsatellite stable
MSI: Microsatellite instability
MS-PCR: Methylation Specific-PCR
NGS: Next generation sequencing
NPY: Neuropeptide Y
ROC: Receiver operatoring characteristic
WIF1: Wnt inhibitory factor 1
