# Supplementary material for: The detection of specific hypermethylated WIF1 and NPY genes in circulating DNA by crystal digital PCR™ is a powerful new tool for colorectal cancer diagnosis and screening
Source: BMC Cancer. 2021 Oct 10;21:1092. doi: 10.1186/s12885-021-08816-2 (PMC8502418; doi:10.1186/s12885-021-08816-2)
Supplement: Supplementary file 1 — Additional file 1. [file 12885_2021_8816_MOESM1_ESM.doc]

SUPPLEMENTARY DATA

**Supplementary data 1**

Demography of 10 patients’ ctDNA in the control group (sex, ages, oncologic background). The sex repartition was equal. The mean age was 49 years old (min: 21y – max: 65y).

| **N° patient** | **Age (years)** | **Sex** | **Positive droplets** | **Oncologic background** |
| --- | --- | --- | --- | --- |
| **1** | **35** | **M** | **0** | **None** |
| **2** | **36** | **M** | **0** | **None** |
| **3** | **65** | **F** | **0** | **None** |
| **4** | **24** | **F** | **0** | **None** |
| **5** | **63** | **M** | **0** | **None** |
| **6** | **60** | **F** | **0** | **None** |
| **7** | **64** | **M** | **0** | **None** |
| **8** | **63** | **F** | **0** | **None** |
| **9** | **59** | **M** | **0** | **None** |
| **10** | **21** | **F** | **0** | **None** |

*F: Female; M: Male.*


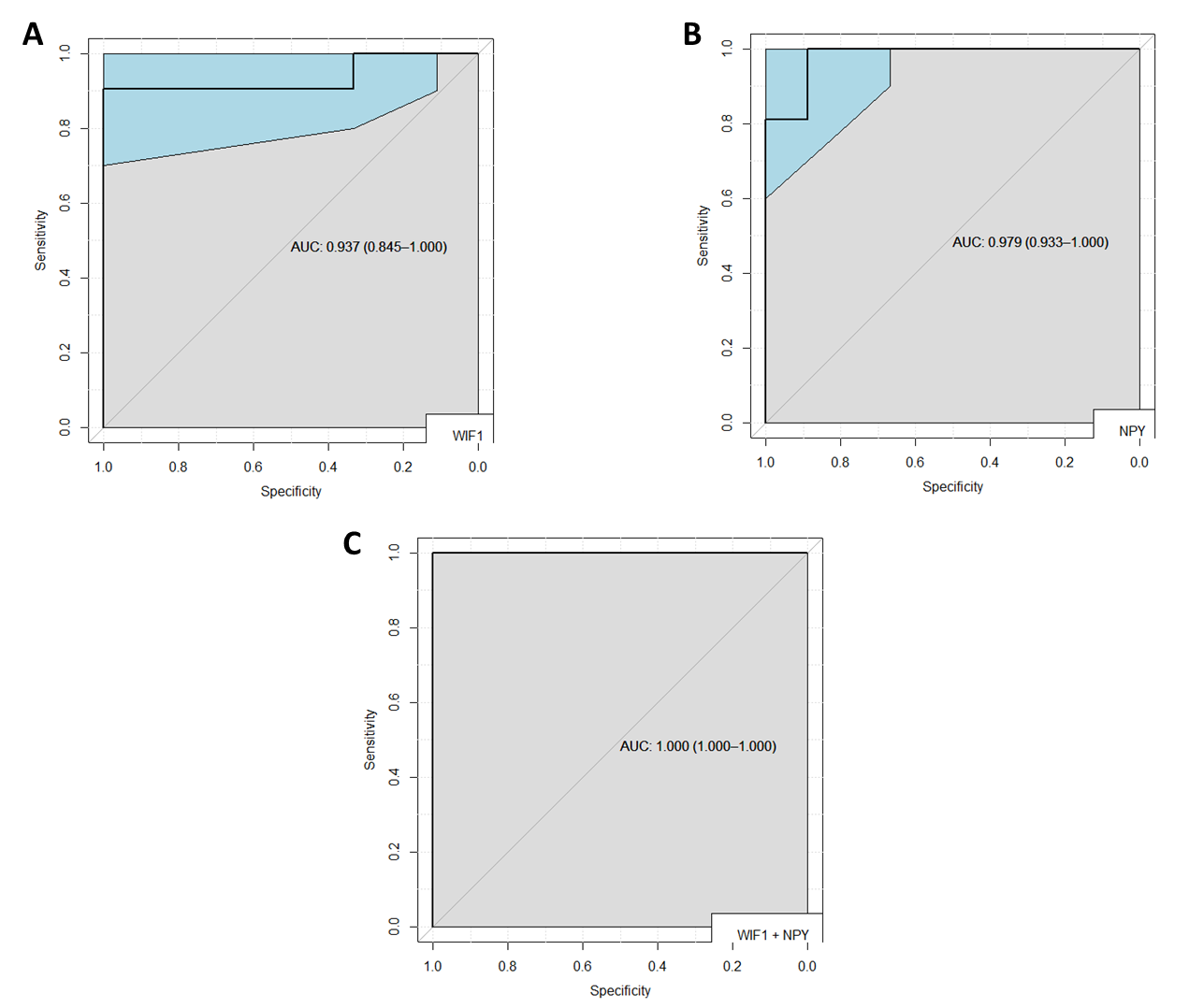


**Supplementary data 2: ROC curves comparing non-tumor vs tumor according to *WIF1* or/and *NPY* methylation.**

Receiver operating characteristic (ROC) curve of (A) *NPY*alone, (B) *WIF1*alone and (C) the combination of *NPY*and *WIF1*on the local colorectal tissue samples*.*

A ROC curve shows the performance of a quantitative test at all the possible thresholds, the area under the ROC curve (AUC) is a global evaluation of the classification performance of a quantitative test. The combination of *NPY* and *WIF1* by addition of the positive droplets increases the performance of the test, with a 100% sensitivity and specificity.


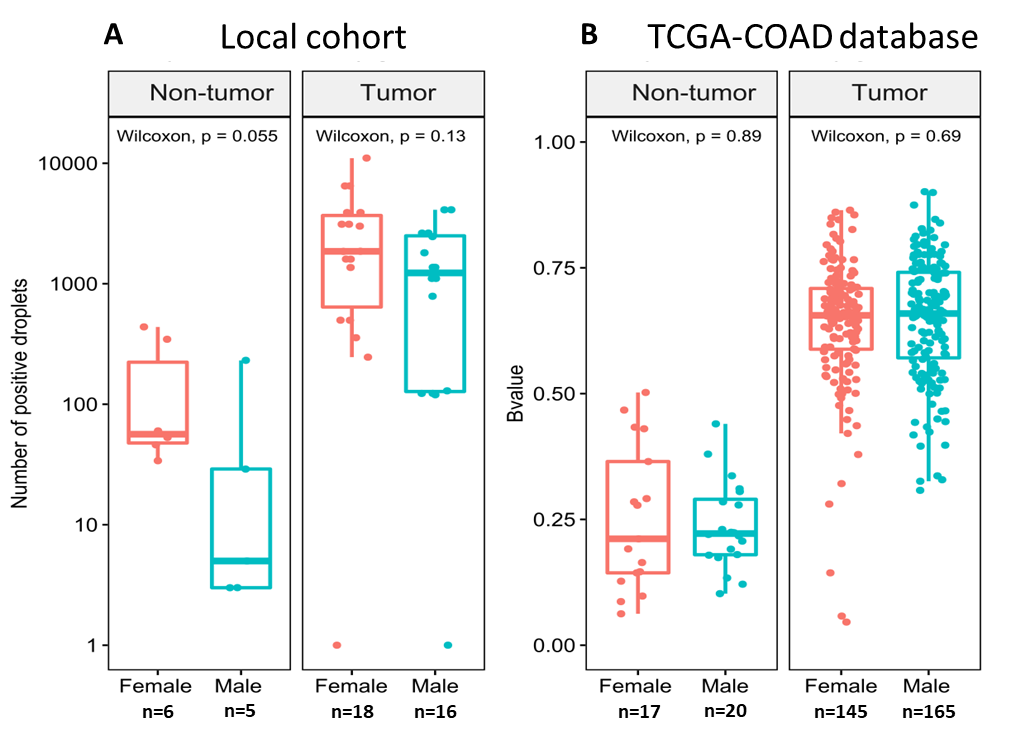


**Supplementary data 3:** **Methylation of NPY according to gender**

(A) Boxplot of the number of positive droplets in tissue samples in the local cohort grouped by gender and tissue type. (B) Boxplot of the Bvalue in tissue samples in TCGA-COAD grouped by gender and tissue type. Bvalue represent the percentage of methylation of the promoter of *NPY* in a sample.

No significant differences according to gender were found in the local cohort nor in TCGA data between the colorectal tissue samples.
